# Resistance Analyses of HCV NS3/4A Protease and NS5B Polymerase from Clinical Studies of Deleobuvir and Faldaprevir

**DOI:** 10.1371/journal.pone.0160668

**Published:** 2016-08-05

**Authors:** Kristi L. Berger, Christoph Sarrazin, David R. Nelson, Joseph Scherer, Nanshi Sha, Martin Marquis, Alexandra Côté-Martin, Richard Vinisko, Jerry O. Stern, Federico J. Mensa, George Kukolj

**Affiliations:** 1 Boehringer Ingelheim Pharmaceuticals Inc., Ridgefield, CT, United States of America; 2 Boehringer Ingelheim Ltd/Ltée, R&D, Laval, QC, Canada; 3 J.W. Goethe University Hospital, Frankfurt, Germany; 4 Clinical and Translational Science Institute, University of Florida, Gainesville, FL, United States of America; 5 Boehringer Ingelheim Ltd/Ltée, Burlington, ON, Canada; University of Cincinnati College of Medicine, UNITED STATES

## Abstract

**Background & Aim:**

The resistance profile of anti-hepatitis C virus (HCV) agents used in combination is important to guide optimal treatment regimens. We evaluated baseline and treatment-emergent NS3/4A and NS5B amino-acid variants among HCV genotype (GT)-1a and -1b-infected patients treated with faldaprevir (HCV protease inhibitor), deleobuvir (HCV polymerase non-nucleoside inhibitor), and ribavirin in multiple clinical studies.

**Methods:**

HCV NS3/4A and NS5B population sequencing (Sanger method) was performed on all baseline plasma samples (n = 1425 NS3; n = 1556 NS5B) and on post-baseline plasma samples from patients with virologic failure (n = 113 GT-1a; n = 221 GT-1b). Persistence and time to loss of resistance-associated variants (RAVs) was estimated using Kaplan–Meier analysis.

**Results:**

Faldaprevir RAVs (NS3 R155 and D168) and deleobuvir RAVs (NS5B 495 and 496) were rare (<1%) at baseline. Virologic response to faldaprevir/deleobuvir/ribavirin was not compromised by common baseline NS3 polymorphisms (e.g. Q80K in 17.5% of GT-1a) or by NS5B A421V, present in 20% of GT-1a. In GT-1b, alanine at NS5B codon 499 (present in 15% of baseline sequences) was associated with reduced response. Treatment-emergent RAVs consolidated previous findings: NS3 R155 and D168 were key faldaprevir RAVs; NS5B A421 and P495 were key deleobuvir RAVs. Among on-treatment virologic breakthroughs, RAVs emerged in both NS3 and NS5B (>90%). Virologic relapse was associated with RAVs in both NS3 and NS5B (53% GT-1b; 52% GT-1b); some virologic relapses had NS3 RAVs only (47% GT-1a; 17% GT-1b). Median time to loss of GT-1b NS5B P495 RAVs post-treatment (5 months) was less than that of GT-1b NS3 D168 (8.5 months) and GT-1a R155 RAVs (11.5 months).

**Conclusion:**

Faldaprevir and deleobuvir RAVs are more prevalent among virologic failures than at baseline. Treatment response was not compromised by common NS3 polymorphisms; however, alanine at NS5B amino acid 499 at baseline (wild-type in GT-1a, polymorphism in GT-1b) may reduce response to this deleobuvir-based regimen.

## Introduction

The management of patients with hepatitis C virus (HCV) genotype (GT)-1 infection has been transformed over recent years with the introduction of oral direct-acting antivirals (DAAs) that target essential HCV encoded viral functions [1]. Because of the limitations of interferon (IFN)-based regimens, attention has been focused on combining multiple DAAs that target different viral functions in IFN-free treatment regimens, some of which are now in clinical use [1].

Faldaprevir is a HCV NS3/4A protease inhibitor (PI) with potent *in vitro* activity against HCV GT-1a and -1b, and a pharmacokinetic profile that supports once-daily (QD) dosing [2, 3]. Deleobuvir is a non-nucleoside inhibitor (NNI) of HCV NS5B RNA polymerase that binds reversibly to thumb-pocket 1 of NS5B [4, 5]. The IFN-free combination of faldaprevir QD, plus deleobuvir twice daily or three-times daily, with or without ribavirin (RBV) was investigated in phase 2 and 3 clinical studies in treatment-naïve patients with chronic HCV GT-1 infection [6–10]. In phase 2 studies, the rate of sustained virologic response 12 weeks after the end of treatment (SVR12) was higher for HCV GT-1b than for GT-1a-infected patients (particularly GT-1a-infected patients with *IL28B* non-CC genotypes) [9, 10]. Phase 3 studies (HCVerso1 and 2) assessed 16- and 24-week treatment durations in treatment-naïve, HCV GT-1b-infected patients, including patients ineligible for treatment with pegylated (Peg) IFN (HCVerso2). SVR12 rates were 72–83% among patients without cirrhosis and 73–74% among patients with compensated cirrhosis [7, 8].

Resistance to faldaprevir has been extensively studied both *in vitro* and in clinical studies [2, 11–13]. The emergence of faldaprevir resistance-associated variants (RAVs) is characterized by amino acid substitutions in the inhibitor binding pocket of the NS3 protease, highlighted by residues R155K in GT-1a and D168V in GT-1b isolates. *In vitro* studies and phase 1b clinical studies of deleobuvir show the emergence of RAVs in the thumb-pocket 1 binding site, predominantly NS5B P495L variants [4, 14, 15]. Combining antiviral agents acting on different targets raises the genetic barrier to resistance. Understanding the resistance profile of DAAs used in combination is important to guide selection of optimal combinations for first-line therapy and for the effective re-treatment after failure to respond to first-line therapy. This is particularly important for novel classes of HCV DAAs that include NS3 protease inhibitors and NNI compounds such as deleobuvir, which was the first NS5B thumb-pocket 1 inhibitor to progress to phase 3 trials and has been recently followed by beclabuvir (BMS-791325) and TMC-647055 [16, 17].

We performed a comprehensive analysis of HCV NS3/4A and NS5B baseline polymorphisms and treatment-emergent RAVs detected in samples from patients receiving combinations of faldaprevir and deleobuvir in phase 2 and 3 clinical studies. We aimed to assess the impact of baseline NS3/4A and NS5B polymorphisms on the virologic response to treatment, to identify and characterize treatment-emergent RAVs, and to estimate their persistence during post-treatment follow-up.

## Materials and Methods

### Study design and HCV sequences

Resistance analyses were performed on plasma virus samples derived from HCV GT-1-infected patients treated with deleobuvir (with or without PegIFN/RBV) in two phase 1b studies (ClinicalTrials.gov NCT02176525 and NCT00905632) or from patients treated with faldaprevir plus deleobuvir (with or without RBV) in a phase 1b (Clinicaltrials.gov NCT01132313 SOUND C1), two phase 2b (Clinicaltrials.gov NCT01132313 SOUND C2 and C3), and two phase 3 (ClinicalTrials.gov NCT01732796 and NCT01728324) studies that were performed in Europe, North America, Australia, and New Zealand. The individual studies have been described in detail elsewhere and are summarized in **Table A in S1 Supplementary Data** [4, 6–10, 15]. In all the studies, patients with HIV or HBV con-infection were excluded. The numbers of GT-1a and GT-1b-infected patients with HCV NS3/4A and NS5B population-based sequences are given in **Tables B and C in S1 Supplementary Data**. Nearly all patients (>99%) had baseline virus genotypic data. Among HCV-infected patients who did not achieve SVR12 with faldaprevir/deleobuvir/RBV treatment (SOUND-C2, SOUND-C3, and HCVerso studies), matched baseline and post-baseline NS3/4A and NS5B sequences were available for 113 GT-1a-infected patients and for 217 (NS3/4A) and 219 (NS5B) GT-1b-infected patients.

The study was approved by the institutional review board/independent ethics committee of each participating site, and was carried out in compliance with the ethical guidelines of the Declaration of Helsinki and in accordance with the International Conference on Harmonisation Guidelines for Good Clinical Practice. All patients provided written informed consent prior to enrolment.

### Virologic responses

Plasma HCV RNA was measured using COBAS TaqMan HCV/HPS v2.0. The primary efficacy endpoint of phase 2 and 3 studies was SVR12 (HCV RNA <25 IU/mL 12 weeks after the last planned dose of study drug). Treatment failure was categorized as: virologic breakthrough on-treatment (an increase in HCV RNA of ≥1 log_10_ from nadir or HCV RNA ≥25 IU/mL following an earlier decrease to <25 IU/mL, confirmed by a second consecutive measurement of ≥25 IU/mL within a 2-week duration) or relapse (HCV RNA >25 IU/mL in patients who had undetectable HCV RNA at the end of treatment). Other reasons for lack of SVR12 included: null response (phase 2, detectable HCV RNA at weeks 6 and 8; phase 3, failure to achieve HCV RNA <25 IU/mL and HCV RNA ≥25 IU/mL at any time after week 8); premature discontinuation for non-virologic reasons (phase 2 and 3; excludes discontinuation during placebo treatment in phase 3); lack of end of treatment response (phase 2 and 3); and lost to follow-up or missing SVR12 data (phase 2 and 3).

### Resistance analysis

Population sequencing of NS3/4A (NS3 amino acids 1–631, NS4A amino acids 1–54) and NS5B (amino acids 1–591) was performed by Boehringer Ingelheim (Canada), R&D, Ltd. (Laval, Quebec) using BigDye Terminator version 3.1 (Applied Biosystems) and an ABI Prism 3130XL Genetic Analyzer (Applied Biosystems), as previously described [4, 13] or by DDL Diagnostic Laboratory (Rijswijk, The Netherlands). The resulting nucleotide sequences were analyzed with SeqScape version 2.5 (Applied Biosystems). All baseline plasma samples were sequenced, and post-baseline sequencing was performed on virologic rebound samples with HCV RNA ≥1000 IU/mL or on samples in which the HCV RNA plateaued above 1000 IU/mL. Virologic rebound samples that were not analyzed included those with HCV RNA that failed to amplify and generally had plasma HCV RNA below the lower limit of amplification (1000 IU/mL). For patients with treatment-emergent RAVs, subsequent post-treatment follow-up plasma samples were sequenced to assess RAV persistence.

Nucleotide and amino acid sequences were compared with GT-1 references: H77 for GT-1a (GenBank accession #AF009606) and Con-1 for GT-1b (GenBank accession #AJ238799). Amino acid positions of interest included: NS3 codons 61, 155, 156, and 168 (based on previous reports showing emergence during faldaprevir treatment); NS3 codons 80 (associated with reduced response to simeprevir) and 344 (associated with reduced response to faldaprevir plus PegIFN/RBV and to placebo plus PegIFN/RBV); and NS5B codons 495, 496, and 499 (deleobuvir-associated resistance) [1, 4, 11, 12, 15, 18]. GenBank accession numbers for baseline sequences from phase 1b, 2, and 3 studies are KT870159—KT871583 for NS3/4A and KT871584—KT873139 for NS5B.

NS3/4A and NS5B *in vitro* phenotyping and drug sensitivity assays were performed as previously described [4, 13]. Additional details of genotyping and phenotyping methods are provided in **S1 Supplementary Data**.

### Statistical analysis

Percentages of patients who achieved SVR12 with and without baseline amino acid variants were compared using a two-sided Fisher’s exact test; phase 2 and 3 datasets included patients with breakthrough, lack of end-of-treatment response, relapse, or SVR12; and phase 2 datasets also included all other reasons for lack of SVR12. Comparisons of baseline susceptibility to deleobuvir, for clinical isolates with and without a particular NS5B amino acid variant, used a two-sided Wilcoxon test. ‘With variant’ included only the specific variant and not amino acid mixtures. ‘Without variant’ included wild-type and all other amino acid variants or mixtures.

Long-term persistence and median time to loss of RAVs (any variants detected at individual NS3 R155, NS3 D168, or NS5B P495 codons) with outgrowth of wild-type virus was estimated using survival curve analysis in GraphPad Prism v6.05 based on the Kaplan–Meier method (see **S1 Supplementary Data**). Comparisons of survival curves were performed using a log-rank (Mantel–Cox) test with GraphPad Prism v6.05.

All tests conducted were considered descriptive to provide an assessment of the degree of effect observed. Thus, no alpha adjustments were made for multiple comparisons.

## Results

### NS3/4A and NS5B baseline polymorphisms

The prevalence of baseline amino acid polymorphisms (key positions defined in Materials and Methods) is shown in **Table 1**. Faldaprevir RAVs at NS3 R155 or D168 were rare (<1%) at baseline, and A156 polymorphisms were not detected in either genotype. NS3 Q80K was detected in 17.5% of GT-1a samples. NS3 T/S61L amino acid substitutions were not observed. The NS3 helicase T344I polymorphism (I or I/T mixture) was detected in approximately 21% of GT-1b baselines, but was not detected in GT-1a. Deleobuvir RAVs at NS5B P495 or P496 were rare or not observed. NS5B A421V was a common polymorphism in GT-1a (20.2%). NS5B polymorphisms at codon 499 were common in GT-1b, including 14.8% with V499A (A or A/V mixtures) or 5.5% with V499T. The subtype specific wild-type alanine (A) at position 499 in GT-1a was 96.6% conserved.

**Table 1:** Prevalence of baseline HCV NS3/4A and NS5B polymorphisms.

Codon	Reference (%)	Variants		Reference (%)	Variants	
		≥1% (%)	<1%		≥1% (%)	<1%
**NS3/4A**	**HCV GT-1a (n = 229)**			**HCV GT-1b (n = 1196)**		
**61**	T (93.0)	A, A/T (4.4)	-	S (85.2)^a^	T, S/T (7.4)	A/S/T, A/T, C, N
		S, S/T (2.6)			A, A/S (4.5)	
					P, P/S (2.4)	
**80**	Q (80.8)	K, K/Q, K/N (17.5)	R, L/Q, I/K/L/Q	Q (96.5)	L, L/Q (2.4)	K, K/Q, L/M, R
**155**	R (99.1)	-	K	R (100)	-	-
**156**	A (100)	-	-	A (100)	-	-
**168**	D (100)	-	-	D (99.0)	E, D/E (1.0)	-
**344**	T (99.1)	-	A/T, N	T (67.3)	I, I/T (20.8)	I/V, D, D/V, D/I/N/V, A/I/T/V, A/S, I/M, I/M/T, N/S, N/T
					V (6.9)	
					M, M/T (1.3)	
					S, S/T (1.2)	
					A, A/T (1.0)	
**NS5B**	**HCV GT-1a (n = 292)**			**HCV GT-1b (n = 1196)**		
**421**	A (79.5)	V, A/V (20.2)	E/V	A (91.9)	V, A/V (8.0)	G, T
**495**	P (100)	-	-	P (100)	-	-
**496**	P (100)	-	-	P (99.5)	-	A, S, P/S
**499**	A (96.6)	T (2.7)	A/S, V	V (78.5)	A, A/V (14.8) T (5.5)	A/T, I, I/V, L

Pooled data: 1241.2, 1241.7, SOUND-C1, -C2, and -C3, and HCVerso1 and 2 (all treatment arms).

^a^Denominator was 1195 due to one illegible sequence at NS3 codon 61.

GT, genotype; HCV, hepatitis C virus.

**Fig 1:**
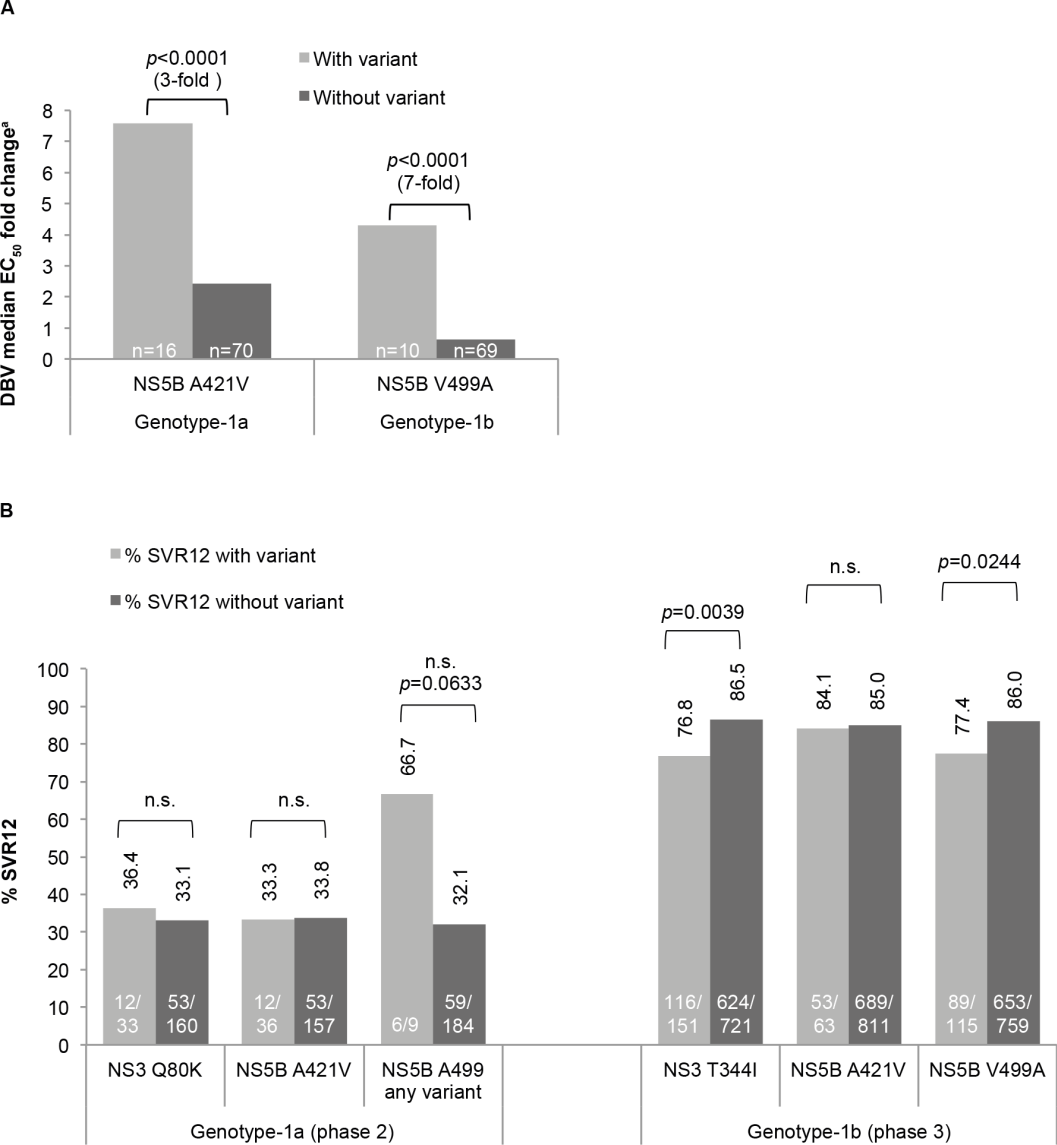
**Impact of baseline HCV polymorphisms on (A) *in vitro* deleobuvir susceptibility and on (B) response to treatment with faldaprevir/deleobuvir/RBV**. Data pooled from (A) 1241.2, 1241.7, SOUND-C1, and SOUND-C2; and (B) SOUND-C2 (RBV-containing arms only), SOUND-C3 (phase 2 data), and HCVerso1 and 2 (phase 3 data). Statistical analyses are described in ‘Materials and Methods’. GT-1a ‘NS5B A499 any variant’ includes A499T (n = 4), A499A/S, and A499V. ^a^Fold change with respect to subtype reference EC_50_ of sub-genomic replicons (GT-1a = 17.5 nM [n = 19] and GT-1b = 11.5 nM [n = 49]). DBV, deleobuvir; EC_50_, 50% effective concentration; HCV, hepatitis C virus; n.s., not significant (*p*>0.05); RBV, ribavirin; SVR12, sustained virologic response 12 weeks after treatment.

Phenotypic evaluation of chimeric HCV sub-genomic replicons containing NS5B polymerases derived from baseline isolates showed that the only common polymorphisms associated with reduced *in vitro* susceptibility (*p*<0.0001) to deleobuvir were GT-1a A421V and GT-1b V499A (**Fig 1A**). *In vitro* faldaprevir susceptibility of patient-derived NS3 proteases has been previously described [13].

Baseline GT-1a NS3 Q80K had no significant impact on SVR12 following treatment with faldaprevir/deleobuvir/RBV (**Fig 1B**, pooled phase 2 studies). The GT-1b NS3 helicase T344I baseline polymorphism was associated with a lower SVR12 rate in phase 3 studies (76.8% [116/151] with T344I, 86.5% [624/721] without T344I; **Fig 1B**), but not in phase 2 studies (**Table D in S1 Supplementary Data**).

SVR12 rates among patients with GT-1a or GT-1b infection were similar with or without baseline NS5B A421V (**Fig 1B**). The GT-1b NS5B V499A baseline polymorphism was associated with a reduced SVR12 rate in phase 3 studies (77.4% [89/115] with V499A, 86% [653/759] without V499A; **Fig 1B**), but not in phase 2 studies (**Table D in S1 Supplementary Data**). Although the sample size was small, a higher SVR12 rate was noted among the few patients with GT-1a NS5B 499 variants than with wild-type A499 (**Fig 1B**).

### Treatment-emergent NS3/4A and NS5B variants

Most patients who did not achieve SVR12 in phase 2 and 3 studies following faldaprevir/deleobuvir/RBV treatment had RAVs emerge at NS3 155, NS3 168, and/or NS5B 495 RAVs with or without NS5B A421V (**Table 2**). The most common RAVs were NS3 R155K and NS5B P495L in GT-1a, and among GT-1b were NS3 D168V, NS5B P495L, and NS3 R155 RAVs, and included other variants at these positions.

**Table 2:** Treatment-emergent NS3 and NS5B substitutions in patients who did not achieve SVR12 with faldaprevir/deleobuvir/RBV.

Frequency of amino acid variants^a^ (%)	HCV GT-1a		HCV GT-1b	
	NS3/4A (n = 113)	NS5B (n = 113)	NS3/4A (n = 216)	NS5B (n = 218)
**≥10% of non-SVR12 patients**	Any R155 (94.7%)	Any P495 (77.0%)	Any D168 (57.4%)	Any P495 (59.4%)
	R155K (93.8%)	P495L (72.6%)	Any R155 (18.5%)	P495L (39.7%)
		A421V^b^ (19.5%)	D168V (32.4%)	
**1% to <10% of non-SVR12 patients**	Any D168 (6.2%)	P495S (8.8%)	D168N (8.8%)	P495Q (8.7%)
	D168V (3.5%)	P495T (3.5%)	R155Q (8.3%)	P495T (7.3%)
	D168E/N (1.8%)	P496S (2.7%)	D168T (7.9%)	P495S (6.4%)
	R155T^c^ (1.8%)		D168E (6.9%)	A421V^b^ (4.6%)
			R155G^c^ (6.5%)	V499A (2.7%)
			A156T^d^ (4.2%)	P496S (1.4%)
			R155K (3.7%)	
			D168A (3.2%)	
			D168H/I (1.8%)	
			R155W (1.8%)	
			S61L^e^ (1.4%)	
**<1% of non-SVR12 patients**	R155G/S^f^, A156P/S/T^f^		R155S, D168Y, A156V^d^, D168G/S^e^	P496A, P496L
	D168 ambiguous mixture with V	P495 ambiguous mixtures with L	R155 ambiguous mixture with K	P495 ambiguous mixture with L

Pooled data: SOUND-C2 (RBV-containing arms only), SOUND-C3, and HCVerso1 and 2.

^a^Amino acids variants detected alone or in combination with other substitutions (includes mixtures).

^b^Detected with P495L variants in 15.0% of GT-1a and 3.7% of GT-1b.

^c^Commonly detected as a mixture with D168N.

^d^Only detected in phase 3 studies with NS5B P495L/T or P496S RAVs and without NS3 R155 or D158 RAVs.

^e^Only detected with D168 variants; denominator was n = 213 due to illegible sequence at NS3 codon 61 for 3 patients.

^f^Only detected in mixture of amino acid substitutions as listed.

GT, genotype; HCV, hepatitis C virus; RAV, resistance-associated variant; RBV, ribavirin; SVR12, sustained virologic response 12 weeks after treatment.

NS5B A421V emerged more frequently in GT-1a (19.5% overall) than in GT-1b (4.6% overall) and often as a co-variant with NS5B P495L among virologic breakthroughs. NS5B A421V+P495L dual RAVs were detected in 15% (17/113) of GT-1a non-SVR patients (15/17 were breakthroughs) and in 3.7% (8/219) of GT-1b non-SVR patients (7/8 were breakthroughs).

Variants at NS3 61, NS3 156, NS5B 496, and NS5B 499 emerged at a low frequency. The GT-1b NS3 S61L co-variant emerged consistently with D168 RAVs (three virologic breakthroughs and one patient without SVR for other reasons). Overall, NS3 A156T/V variants were detected in 4.6% (10/216) of HCV GT-1b samples and only in the phase 3 studies (six patients with virologic breakthrough, three with relapse, and one without SVR12 for other reasons). These variants always emerged with NS5B P495L/T or P496S RAVs and without NS3 R155 or D168 RAVs. Mixed amino acid variants at position 156 emerged in only one GT-1a patient in phase 2 studies (A156P/S/T). NS5B RAVs at position 496 also emerged in HCV GT-1a (P496S in one patient with breakthrough and two without SVR12 for other reasons) and HCV GT-1b (P496S in three patients without SVR12 for other reasons, P496A and P496L each in one with relapse). NS5B V499A emerged in 2.7% of GT-1b non-SVR patients and only in phase 3 studies (two breakthroughs, three relapses, and one ‘other’ reason for non-SVR).

Treatment-emergent variants at NS5B codons 389, 415, and 390 or at GT-1b NS3 codon 344 were evaluated as described in **S1 Supplementary Data**. Variants at these positions were infrequent and not attributable specifically to combination therapy with faldaprevir/deleobuvir/RBV (**Table E in S1 Supplementary Data**).

### Treatment-emergent RAVs in single or multiple target genes

**Fig 2:**
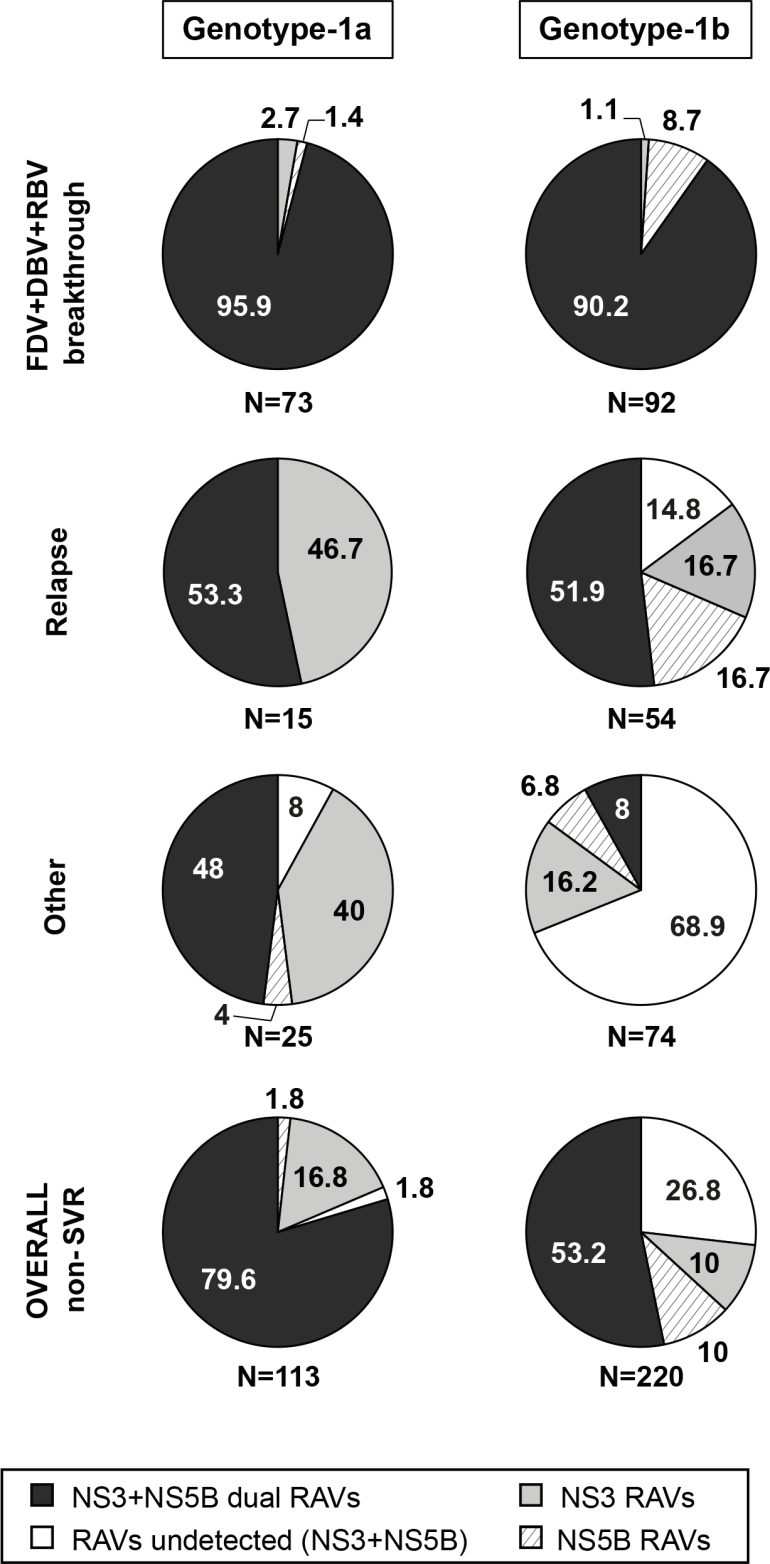
Treatment-emergent RAVs in NS3, NS5B, or both among patients who did not achieve SVR12 with faldaprevir/deleobuvir/RBV. Pooled data: SOUND-C2 (RBV-containing arms only), SOUND-C3, and HCVerso1 and 2. Only patients with sequence data available are shown. NS3 RAV: R155 and/or D168 variants with or without S61L, or single A156 variants. NS5B RAV: P495, P496, A421V, and/or V499A variants. Reasons for non-SVR12 are described in ‘Materials and Methods’. DBV, deleobuvir; FDV, faldaprevir; RAV, resistance-associated variant; RBV, ribavirin; SVR12, sustained virologic response 12 weeks after treatment.

**Fig 2** shows the association of RAVs in NS3 and NS5B among patients categorized according to reason for failure to achieve SVR12 among n = 113 GT-1a and n = 220 GT-1b-infected patients; HCV sequence data was provided for at least one or both genes. Overall, dual NS3+NS5B RAVs or RAVS in at least one target gene were detected in 98.2% of GT-1a and 73.3% of GT-1b non-SVR patients. Among patients with virologic breakthrough (GT-1a and HCV GT-1b), at least one treatment-emergent RAV in NS3/4A and/or NS5B was detected in all cases and dual RAVs (i.e. one in NS3 and one in NS5B) were detected in most cases.

For patients with virologic relapse, treatment-emergent dual RAVs were detected in a smaller majority of samples. In 16.7% of HCV GT-1b relapses, only NS5B RAVs were detected. Detection of NS3 RAVs in the absence of NS5B RAVs was more frequent in HCV GT-1a than in HCV GT-1b. Whereas all GT-1a relapses encoded NS5B and/or NS3 RAVs, 14.8% of GT-1b relapses lacked detectable RAVs in both genes.

Among patients who did not achieve SVR12 for other reasons, emergence of dual RAVs was more frequent among GT-1a than among GT-1b samples, a large proportion of which had no RAVs or only NS3 RAVs detected.

### Post-treatment persistence of RAVs

Persistence of NS3 or NS5B variants (assessed by population-based sequencing) during post-treatment follow-up was evaluated in 50 GT-1a-infected (phase 2) and 101 GT-1b-infected patients (phase 3). Patients with virologic failure (breakthrough, relapse, or other reasons), with at least one post-baseline sequence, and who did not receive subsequent PegIFN/RBV rescue therapy, had virologic failure sequences for NS3 and/or NS5B and had additional follow-up virology sequences after the first virologic failure sequence was evaluated. The median follow-up time from the day of virologic failure to the last viral NS3 and/or NS5B sequence available was 244 days (range 12–693 days) for GT-1a-infected patients and 149 days (range 28–406 days) for GT-1b-infected patients.

**Fig 3:**
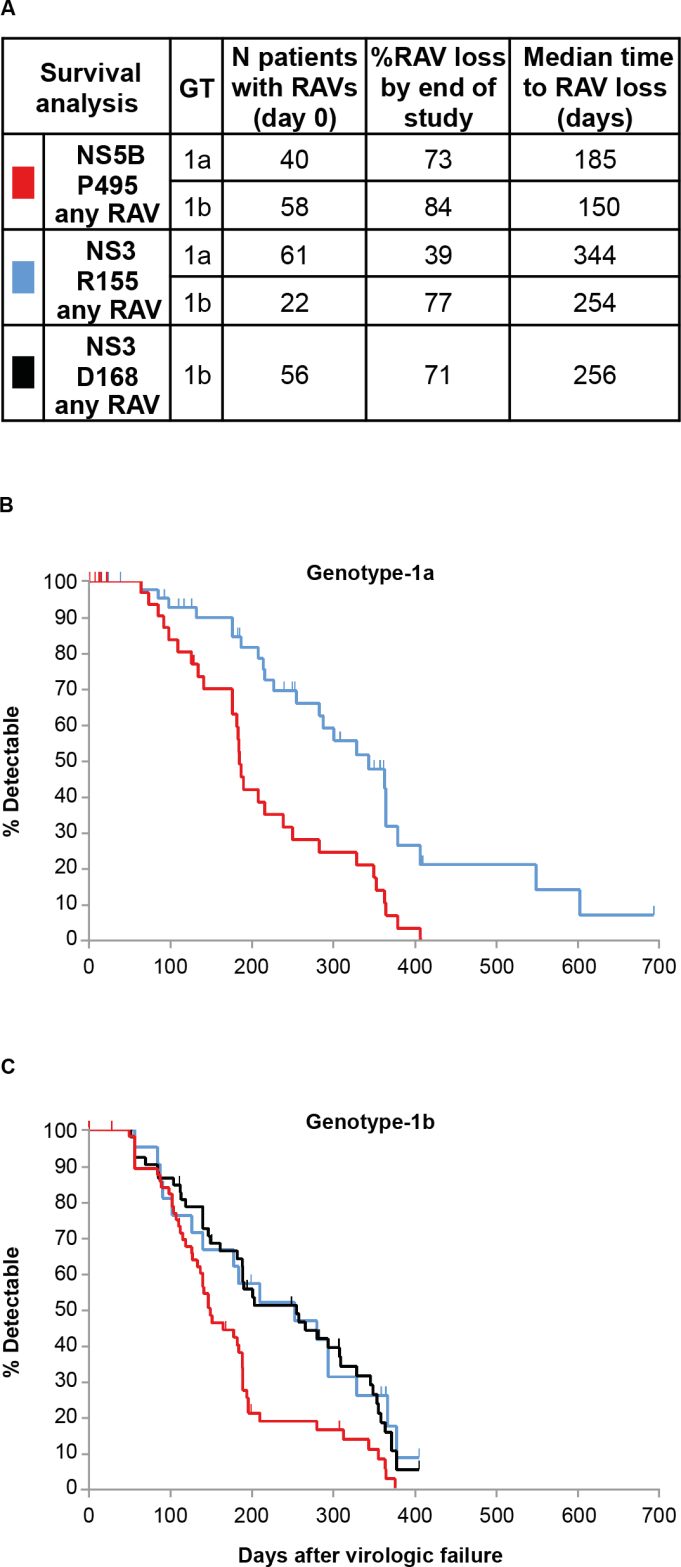
Persistence of NS3 and NS5B RAVs among patients who did not achieve SVR12 with faldaprevir/deleobuvir/RBV and did not receive PegIFN/RBV rescue therapy. (A) Kaplan–Meier estimation of time to loss of RAVs (population-based sequencing); (B) Phase 2 studies SOUND-C2 and SOUND-C3. (C) Phase 3 studies HCVerso1 and 2. Median time to RAV loss: time to wild-type amino acid detection (with concomitant loss of RAVs) in 50% of patients. Vertical marks: RAV detected, but subsequent sequence not available. GT, genotype; PegIFN, pegylated interferon; RAV, resistance-associated variant; RBV, ribavirin; SVR12, sustained virologic response 12 weeks after treatment.

Among GT-1a-infected patients, the median time to loss of RAVs and outgrowth of wild-type virus was longer for NS3 R155 RAVs (n = 61, 344 days; ~11.5 months) than for NS5B P495 RAVs (n = 40, 185 days; ~6 months; **Fig 3A and 3B**). The median time to loss of GT-1b NS3 D168 RAVs (n = 56 with any variant) with outgrowth of wild-type (~8.5 months) was longer than the median time to loss of GT-1b NS5B P495 RAVs (n = 58 with any variant; ~5 months; **Fig 3A and 3C**). Median time to loss of GT-1b NS3 R155 RAVs was similar to the time to loss of GT-1b NS3 D168 RAVs. The decline of GT-1a NS5B P495 detected RAV was significantly more rapid than the decline of GT-1a NS3 R155 detected RAV (*p* = 0.0002). Similarly, the decline of GT-1b NS5B P495 detected RAV was significantly more rapid than the decline of NS3 D168 RAVs (*p* = 0.0028). Survival curves for NS5B P495 RAVs in either GT-1a or GT-1b were not significantly different (*p* = 0.1202).

### Phenotypic characterization of RAVs

Single and dual treatment-emergent NS3/4A or NS5B amino acid substitutions detected in HCV from patients without SVR12 in clinical studies were introduced into GT-1a and GT-1b replicon backgrounds by site-directed mutagenesis and the replicative capacity, as well as the faldaprevir or deleobuvir susceptibility, was assessed in phenotypic assays (**Table 3 and Tables H and I in S1 Supplementary Data**). The NS5B A421V mutant conferred only a 3-fold reduction in sensitivity to deleobuvir in both GT-1a and GT-1b NS5B. The highest levels of deleobuvir resistance resulted from the dual NS5B variant A421V+P495L in both GT-1a (150-fold) and GT-1b (1300-fold). The fold change in deleobuvir inhibition of the dual variant was notably higher than with the individual P495L mutant (47-fold for GT-1a and 640-fold for GT-1b). However, the A421V+P495L combination appeared to compromise replicative capacity more than either of the individual substitutions.

**Table 3:** Deleobuvir susceptibility of NS5B site-directed P495L mutants *in vitro*.

	HCV GT-1a		HCV GT-1b	
Site-directed mutant	%RC ± SD (n)	Deleobuvir EC_50_ FC ± SD (n)	%RC ± SD (n)	Deleobuvir EC_50_ FC ± SD (n)
A421V	21 ± 4 (7)	3.2 ± 0.6 (4)	46 ± 14 (10)	2.5 ± 0.6 (6)
P495L	33 ± 26 (13)	47 ± 15 (9)	12 ± 4 (5)	640 ± 140 (4)
A421V+P495L	9.6 ± 1.6 (7)	150 ± 53 (4)	6.6 ± 2.2 (10)	1300 ± 460 (3)

Mean %RC and EC_50_ FC calculated from multiple (n) intra-experimental values relative to wild-type comparator.

EC_50_, 50% effective concentration; FC, fold-change relative to wild-type; GT, genotype; HCV, hepatitis C virus; RC, replicative capacity (% of wild-type); SD, standard deviation.

Phenotypic analyses of patient-derived NS5B domains in replicon assays yielded results that were largely consistent with the results of phenotypic analyses of site-directed mutants. The NS5B A421T substitution alone in a GT-1b isolate had little impact on deleobuvir susceptibility (**Table 4**, patient A). The highest deleobuvir 50% effective concentration (EC_50_) values were reported with the double variant A421V+P495L in two GT-1a isolates (patients B and C). In isolates from patient C, A421V+P495L resulted in an approximately 2-fold greater reduction in deleobuvir susceptibility than P495L alone.

**Table 4:** Deleobuvir susceptibility of novel NS5B variants from patient isolates.

Patient	HCV GT	NS5B variant at rebound	Deleobuvir EC_50_		
			Baseline NS5B (nM)	NS5B at rebound (nM)	Fold shift^a^
A	1b	A421T	37	72	1.9
B	1a	A421V+P495L	97	6367	66^b^
C	1a	P495L	7.5	917	122
		A421V+P495L		2267	302

Data from SOUND-C2.

^a^Fold shift in EC_50_ relative to baseline.

^b^Note higher EC_50_ initially at baseline.

EC_50_, 50% effective concentration; GT, genotype; HCV, hepatitis C virus.

## Discussion

The IFN-free combination of the HCV NS3 PI faldaprevir and the NS5B NNI deleobuvir, with or without RBV, has been assessed in more than 1500 GT-1-infected patients. In phase 2 studies, faldaprevir/deleobuvir/RBV demonstrated higher efficacy in treatment-naïve patients infected with HCV GT-1b than with GT-1a [9, 10]. Factors influencing this lower response rate include the reduced sensitivity of GT-1a NS5B laboratory and clinical isolates to NS5B thumb-pocket 1 inhibitors (including deleobuvir) [4, 5, 17, 19], partly due to GT-1-specific polymorphisms at NS5B codon 499 (predominantly valine in GT-1b and alanine in GT-1a) and a lower barrier to NS3 PI resistance for GT-1a [20]. Notable was the association of baseline NS5B A499 (GT-1a) or V499A (GT-1b) with lower *in vitro* sensitivity and lower virologic response rates in patients infected with HCV GT-1a or GT-1b, supporting the finding that A499 may be associated with lower response in some patients.

The reduced susceptibility to deleobuvir of baseline isolates encoding NS5B polymorphisms A499 or V421 has been previously reported [4] and is supported by the additional phenotyping data collected from phase 1b and phase 2 studies in this report. NS5B A421V is a common GT-1a baseline polymorphism and often emerged as a co-variant with P495L in virologic failures. However, baseline A421V was not associated with reduced SVR. The data also confirmed that baseline NS3 Q80K in HCV GT-1a does not compromise virologic response to faldaprevir-containing treatment regimens [13]. The association of GT-1b NS3 T344I with lower response was not consistent across studies, being observed in phase 3 but not phase 2 studies of faldaprevir/deleobuvir/RBV reported here, and in studies of faldaprevir or placebo plus PegIFN [18], suggesting that the impact of this polymorphism may not be DAA treatment specific.

Overall, the most common emerging variants detected with faldaprevir/deleobuvir/RBV treatment were R155K (GT-1a) or D168 amino acid substitutions (GT-1b) in NS3, and P495L in NS5B (GT-1a and -1b). In most patients with on-treatment virologic breakthrough, HCV RAVs emerged in both target genes (>90% of cases). In contrast, only approximately 50% of patients with relapse after the end of treatment had HCV RAVs in both target genes, whereas the remaining patients with relapse had single NS3 RAVs (GT-1a), RAVs in either NS3 or NS5B, or had wild-type at both loci (GT-1b). The lower frequency of NS5B RAVs detected among relapses suggested that NS5B P495 RAVs may be less fit than NS3 R155 RAVs. Viral clonal sequence analysis from deleobuvir phase 1b studies showed the rapid outgrowth of wild-type NS5B P495 and loss of RAVs in the absence deleobuvir selective pressure [4]. The results of the pooled analysis in this report provide the first assessment of the relative post-treatment persistence of P495 RAVs of an NS3 PI in combination with an NS5B thumb-pocket 1 NNI. The long-term persistence of NS3 R155 RAVs in GT-1a-infected patients is well documented [12, 21]. Here we show that persistence of NS5B P495 RAVs was significantly shorter than NS3 R155 or D168 RAVs RAVs. Notably, the persistence of NS5B P495 RAVs was similar in both GT-1a and GT-1b, whereas NS3 R155 RAVs persisted longer in GT-1a relative to GT-1b. This difference in R155 RAV persistence may reflect the differences in GT1 subtype fitness with these NS3 protease amino acid substitutions. Overall the relative ranking of RAVs, from least to most persistent, was: GT-1a/1b P495 RAVs < GT-1b D168 RAVs ≈ GT-1b R155 RAVs < GT-1a R155 RAVs.

NS3 A156T/V RAVs, identified in pre-clinical faldaprevir studies [11], emerged at a low frequency (4.6% in GT-1b) only among virologic failures that also had NS5B P495 or P496 RAVs in phase 3 deleobuvir studies, and emerged with even rarer frequency in studies of faldaprevir + PegIFN/RBV [18]. Pooled analyses of treatment-emergent amino acid substitutions also identified potential co-variants: NS3 S61L with D168 RAVs in GT-1b and NS5B A421V with P495L typically in GT-1a. The NS3 S61L amino acid substitution was detected more frequently among virologic failures in studies of faldaprevir + PegIFN/RBV that showed that S61L had minimal impact on faldaprevir sensitivity or replication capacity in combination with D168V when characterized *in vitro* [18]. As with faldaprevir/deleobuvir/RBV treatment, NS5B A421V+P495L co-variants were also detected mostly among virologic breakthroughs with beclabuvir (BMS-791325), another NS5B NNI thumb-pocket 1 inhibitor, when combined with PegIFN/RBV therapy [22]. *In vitro* phenotypic studies with either beclabuvir or deleobuvir show that NS5B A421V alone results in only a 3-fold decrease in inhibition; however, the dual NS5B A421V+P495L mutant confers greater resistance than either single mutant alone.

The faldaprevir resistance profile with DAA combination therapy, including deleobuvir plus RBV, is consistent with that observed in other clinical studies of faldaprevir with or without PegIFN/RBV [12, 18]. Faldaprevir shares an overlapping resistance profile with other second-generation protease inhibitors, such as simeprevir, paritaprevir, asunaprevir, and vaniprevir, characterized by GT-1a NS3 R155K RAVs and GT-1b NS3 D168 amino acid substitutions (typically D168V) [23].

NS5B thumb-pocket 1 inhibitors in advanced clinical development include beclabuvir and TMC-647055 [24–27]. In IFN-free DAA-combination studies with these NNI inhibitors, the emergence of NS5B RAVs among virologic failures occurred predominantly at codon P495, which is concordant with deleobuvir clinical studies. Overlapping resistance conferred by NS5B thumb-pocket 1 RAVs at codons 495 (with and without A421V), 496, or 499 is probable among other NNI thumb-pocket 1 inhibitors. However, this resistance profile does not overlap with compounds that inhibit HCV NS5B polymerase by other mechanisms [28]. RAVs associated with NS5B thumb-pocket 2 inhibitors, such as VX-222, include variants at NS5B L419, R422, M423, I482, A486, and V494 [29]. Dasabuvir (ABT-333) is an NS5B palm site 1 inhibitor with an *in vitro* resistance profile including NS5B C316, M414, Y448, and S556 amino acid substitutions [30]. Nucleoside inhibitors (NIs), such as sofosbuvir, provide the highest barrier to resistance in the HCV polymerase because associated NS5B S282T RAVs are rarely detected among virologic failures with sofosbuvir-based therapies [31].

Inhibitors of NS5A have high anti-viral potency in the picomolar range and have been studied in multiple DAA combinations. Regimens consisting of an NS5B thumb-pocket 1 NNI (deleobuvir with RBV, beclabuvir, or TMC-647055) with an NS3 PI and an NS5A inhibitor resulted in higher SVR12 rates in both GT-1a and GT-1b (≥92% SVR12 [24, 27, 32]), compared with studies reported here with two DAAs plus RBV [24, 32]. IFN-free combinations that include the NI sofosbuvir with an NS5A inhibitor (ledipasvir), with or without RBV, can achieve high SVR rates (up to 97% [33]), further highlighting the importance of including an NI and/or NS5A inhibitor in DAA regimens. As HCV anti-viral therapies have advanced rapidly, all IFN-free multi-class drug combinations approved or in phase 3 trials have demonstrated high SVR rates in GT-1-infected patients and contain an NS5A inhibitor with an NS3 PI and an NS5B NNI or an NS5A inhibitor with sofosbuvir [34].

Pooled resistance analyses from phase 2 and 3 studies of faldaprevir/deleobuvir/RBV shows that faldaprevir RAVs at baseline are rare and response to treatment is not compromised by common baseline NS3 polymorphisms; however, GT-1b subtype-specific polymorphisms at NS5B 499 (alanine) may reduce response to this deleobuvir-based two DAA regimen. The emergence of RAVs in patients who did not achieve SVR are consistent with previous findings, which identified NS3 R155 and D168 variants associated with faldaprevir resistance and P495 variants with deleobuvir resistance. Although further development of faldaprevir in combination with deleobuvir was terminated due to a strategic decision, a phase 2 clinical study of faldaprevir in combination with another DAA (NS5A inhibitor) and ribavirin has been initiated (Clinicaltrials.gov Identifier NCT02593162). The studies here provide valuable information about IFN-free HCV antiviral therapies that include a combination of DAAs with a PI and a non-nucleoside thumb-pocket 1 polymerase inhibitor.

## Supporting Information

S1 Supplementary Data (DOCX)
