# Supplementary material for: Resistance Analyses of HCV NS3/4A Protease and NS5B Polymerase from Clinical Studies of Deleobuvir and Faldaprevir
Source: PLoS One. 2016 Aug 5;11(8):e0160668. doi: 10.1371/journal.pone.0160668 (PMC4975400; doi:10.1371/journal.pone.0160668)
Supplement: S1 Supplementary Data — (DOCX) [file pone.0160668.s001.docx]

**Supplementary data to:**

**Resistance analyses of HCV NS3/4A protease and NS5B polymerase from clinical studies of deleobuvir and faldaprevir**

Kristi L Berger^1,2^, Christoph Sarrazin^3^, David R Nelson^4^, Joseph Scherer^1^, Nanshi Sha^1^, Martin Marquis^2^, Alexandra Côté-Martin^1,2^, Richard Vinisko^1^, Jerry O Stern^1^, Federico J Mensa^1^, George Kukolj^2,5^

^1^Boehringer Ingelheim Pharmaceuticals Inc., Ridgefield, CT, USA; ^2^Boehringer Ingelheim Ltd/Ltée, R&D, Laval, QC, Canada; ^3^J.W. Goethe University Hospital, Frankfurt, Germany; ^4^Clinical and Translational Science Institute, University of Florida, Gainesville, FL, USA; ^5^Boehringer Ingelheim Ltd/Ltée, Burlington, ON, Canada

Table of contents

[Supplementary details of methods 2](#_Toc423602704)

[Supplementary results 5](#_Toc423602705)

[Supplementary Table A. Overview of clinical studies included in the analysis. 6](#_Toc423602706)

[Supplementary Table B. Numbers of patients with baseline HCV sequences. 7](#_Toc423602707)

[Supplementary Table C. Numbers of non-SVR12 patients treated with faldaprevir, deleobuvir, and RBV who had post-baseline HCV sequences. 8](#_Toc423602708)

[Supplementary Table D. Impact of baseline polymorphisms on SVR12 rates following treatment with faldaprevir, deleobuvir, and RBV among GT-1b patients in phase 2 studies. 9](#_Toc423602709)

[Supplementary Table E. Treatment-emergent variants at NS5B codons 389, 415, and 390 or GT-1b NS3 codon 344 in patients who did not achieve SVR12 with faldaprevir, deleobuvir, and RBV 10](#_Toc423602710)

[Supplementary Table F. Treatment-emergent RAVs detected in patients without SVR12 following RBV-free treatment with faldaprevir plus deleobuvir in study SOUND-C2. 11](#_Toc423602711)

[Supplementary Table G. Treatment-emergent RAVs detected in samples from patients without SVR24 following treatment with faldaprevir, plus deleobuvir,^a^ and plus RBV followed by faldaprevir + PegIFN/RBV in study SOUND-C1. 12](#_Toc423602712)

[Supplementary Table H. Susceptibility of NS3 site-directed mutants to faldaprevir *in vitro*. 13](#_Toc423602713)

[Supplementary Table I. Susceptibility of NS5B site-directed mutants to deleobuvir *in vitro*. 14](#_Toc423602714)

[Supplemental references 15](#_Toc423602715)

## Supplementary details of methods

*NS3/4A and NS5B population sequencing*

All baseline virology samples were processed for population-based sequencing. Post-baseline sequencing was performed on the first virologic rebound sample with HCV RNA ≥1000 IU/mL or on samples in which the HCV RNA plateaued above 1000 IU/mL. Virologic rebound samples that were not analyzed included those that failed to amplify and generally had plasma HCV RNA below the lower limit of amplification (1000 IU/mL). For patients with treatment-emergent RAVs, subsequent plasma samples during post-treatment follow-up were sequenced to assess the persistence of resistance mutations during the outgrowth of wild-type virus. NS3/4A (amino acids 1–685) and NS5B (amino acids 1–591) population sequencing for phase 1b/2 studies was performed by Boehringer Ingelheim (Canada), R&D, Ltd. (Laval, Quebec) as previously described [1, 2]. For SOUND-C2 and SOUND-C3 studies, all baseline sequences and most first virologic failure samples and follow-up samples were sequenced by Boehringer Ingelheim, Canada. Subsequent sequencing of later first virologic failures or selected follow-up samples from these two trials were completed by DDL Diagnostic Laboratory (Rijswijk, The Netherlands). Population sequencing for phase 3 studies was entirely performed by DDL Diagnostic Laboratory. Mixed nucleotide codons encoding mixed amino acids were assigned when they were represented by at least 10% (Boehringer Ingelheim, Canada) or 25% (DDL Diagnostic Laboratory) of the surface area of the major peak in the DNA electropherogram. All nucleotide and amino acid changes were identified from comparisons to GT-1 reference sequences: H77 for subtype 1a (GenBank accession number AF009606) and Con-1 for subtype 1b (GenBank accession number AJ238799). The few non-GT-1a/1b viral isolates identified are not described here. Two patients from HCVerso1 with GT-1b virus at baseline, but with GT-1a virus at the time of treatment failure, were not included in analyses of virologic response. Resistance analyses of GT-1a isolates summarized here were based only on phase 1b/2 studies because the HCVerso phase 3 analysis plan included only GT-1b isolates; the few GT-1a-infected patients (n=4) from phase 3 were not assessed here.

*NS3 and NS5B phenotyping*

NS3 protease and NS5B phenotyping as well as drug sensitivity assays have been previously described [1, 2]. Briefly, for patient-derived isolates, NS3 or NS5B amplicons were ligated into HCV replicon shuttle vectors containing a luciferase reporter gene to generate chimeric replicons. The reconstituted plasmid DNA was used to generate HCV subgenomic replicon RNA transcripts from this plasmid. These were transfected into Huh-7.5 cells, and luciferase activity was measured as a marker for HCV RNA replication. Serial dilutions of inhibitor were used to determine the EC_50_ for inhibition of HCV RNA replication.

*Post-treatment persistence of RAVs*

Long-term persistence of NS3 or NS5B variants during post-treatment follow-up was evaluated in GT-1a-infected (phase 2) and GT-1b-infected patients (phase 3) with virologic failure (breakthrough, relapse, or other reasons) and at least one post-baseline sequence. Median time to loss of RAVs (any variants detected at individual NS3 R155, NS3 D168, or NS5B P495 codons) with outgrowth of wild-type virus was estimated using survival curve analysis in GraphPad Prism v6.05 based on the Kaplan–Meier method. The analysis included a pooled population of all non-SVR12 patients who (i) were not treated with PegIFN/RBV rescue therapy, (ii) had at least one virologic failure sequence encoding RAVs at the amino acid of interest, and (iii) did not have RAVs at baseline. The time of origin was the date of virologic failure. If a variant was detected after the first date of virologic failure, the variants were imputed back to the failure date. If two consecutive visits had detectable RAVs, the RAVs were imputed for the entire time interval. An ‘event’ was flagged at visits when RAVs were no longer detected due to outgrowth of wild-type. Follow-up virology plasma samples were sequenced only up until the time when outgrowth of wild-type virus was detected (i.e. lack of NS3 and NS5B RAVs). If a RAV was detected at the last visit and no subsequent visits with sequence data were available, then the patient was censored and the RAV was not imputed or carried forward.

The persistence of NS3 D168 RAVs in GT-1a-infected patients was not evaluated because there were few GT-1a-infected patients among virologic failures who did not receive PegIFN/RBV rescue therapy (6.2% [4/65]). There were 65 GT-1a-infected patients and 126 GT-1b-infected patients with virologic failure who did not receive subsequent PegIFN/RBV rescue therapy and had virologic failure sequences for NS3 and/or NS5B. In this subset, the number of non-SVR12 patients who had additional follow-up virology sequences after the first virologic failure sequence (i.e. >1 post-baseline sequence) included 77% (50/65; median 3 sequences) of GT-1a-infected patients and 80% (101/126; median of 2 sequences) of GT-1b patients. Patients who lacked treatment-emergent RAVs and encoded wild-type virus at virologic failure were not evaluated.

## Supplementary results

Treatment-emergent variants at NS5B codons 389, 415, and 390 or GT-1b NS3 codon 344 were infrequent and not attributable specifically to combination therapy with faldaprevir/deleobuvir/RBV (Supplementary **Table E**). NS5B T389S has been reported as a novel escape mutant during *in vitro* drug selection experiments using JT-16, a benzimidazole NNI-1 inhibitor, conferring a 13-fold shift in JT-16 potency [3]. Substitutions at T389 were rare in the clinical studies of deleobuvir included in this report, emerging as a T389I/T mixture in only one GT-1a patient with virologic breakthrough together with the predominant P495L RAV. This indicated that T389 substitutions are not a major component of the deleobuvir clinical resistance profile. NS5B T390I and F415Y may be related to differential sensitivity to RBV [4-6]; however, these substitutions were rarely detected in baseline and post-baseline samples and no association was observed with reduced response rates**.** NS3 T344 variants (A, I, or V) only emerged in GT-1a samples from nine patients.

There was no difference in types or frequency of RAVs between RBV-free and RBV-containing arms in phase 2 studies (Supplementary **Table F**). RAVs emerging after treatment with faldaprevir/deleobuvir/RBV followed by faldaprevir plus PegIFN/RBV in SOUND-C1 are provided in Supplementary **Table G.**

## Supplementary Table A. Overview of clinical studies included in the analysis.

| **Trial  [reference]** | **HCV GT** | **Patient population**  **(N patients)** | | **Treatment** | **Duration** |
| --- | --- | --- | --- | --- | --- |
| **Phase 1b** |  |  | |  |  |
| 1241.2,  NCT02176525 [2] | GT-1a or -1b | Treatment-naïve (16)  Treatment-experienced (57) | | Placebo or  DBV (100–1200 mg TID) | 5 days |
| 1241.7,  NCT00905632 [7] | GT-1a or -1b | Treatment-naïve (27)  Treatment-experienced (30) | | Placebo* or  DBV^#^ (400, 600, or 800 mg) q8h | 28 days |
| **Phase 2 (SOUND-C, NCT01132313)** | | |  |  |  |
| SOUND-C1 [16] | GT-1a or -1b | Treatment-naïve (32) | | FDV 120 mg QD  + DBV (400, or 600 mg TID) + RBV | 0–28 days |
|  |  |  |  | Then, FDV 120 mg QD + PegIFN/RBV | 4–24 or 48 weeks |
| SOUND-C2 [8] | GT-1a or -1b | Treatment-naïve (362) | | FDV 120 mg QD  + DBV (600 mg BID or TID) ± RBV | 16, 28, or 40 weeks |
| SOUND-C3 [9] | GT-1a or -1b^†^ | Treatment-naïve (83) | | FDV 120 mg QD  + DBV 600 mg BID + RBV^†^ | 16 weeks^†^ |
| **Phase 3** |  |  | |  |  |
| HCVerso1,  NCT01732796 [10] | GT-1b | Treatment-naïve (470) | | FDV 120 mg QD + DBV 600 mg TID + RBV | 16 or 24 weeks^‡^ |
| HCVerso2, NCT01728324 [11] | GT-1b | Treatment-naïve (496) | | FDV 120 mg QD + DBV 600 mg TID + RBV | 16 or 24 weeks^‡^ |

Studies were carried out in accordance with the Declaration of Helsinki and International Conference on Harmonisation guidelines. All patients provided written informed consent.

*Only treatment-naïve patients were randomized to the placebo group. ^#^Patients with cirrhosis (n=15) received open-label DBV at 400 or 600 mg q8h. ^†^Patients with IL28B CC genotype and HCV GT-1a infection were assigned to the same treatment as patients with HCV GT-1b infection. Patients with IL28B non-CC genotype and HCV GT-1a infection were randomized to receive FDV 120 mg QD with DBV at 800 mg BID (n=26) or 600 mg TID (n=25), both with RBV, for 24 weeks. ^‡^Patients without cirrhosis were randomized to 16 or 24 weeks of treatment. Patients with compensated cirrhosis all received 24 weeks of treatment (n=51 in HCVerso1 and n=72 in HCVerso2).

BID, twice daily; DBV, deleobuvir; FDV, faldaprevir; GT, genotype; HCV, hepatitis C virus; q8h, every 8 hours; QD, once daily; RBV, ribavirin; TID, three-times daily.

## Supplementary Table B. Numbers of patients with baseline HCV sequences.

| **Trial** | **Trial identifier(s)** | **Patient**  **population** | **GT-1a** | |  | **GT-1b** | |
| --- | --- | --- | --- | --- | --- | --- | --- |
|  |  |  | **NS3/4A^a^ n/N^c^ (%)** | **NS5B^b^  n/N^c^ (%)** |  | **NS3/4A^a^ n/N^c^ (%)** | **NS5B^b^  n/N^c^ (%)** |
| 1241.2^d^  1241.7^e^ | NCT02176525  NCT00905632 | Treatment-naïve  + treatment-experienced | NA | 63/63 (100) |  | NA | 66/66 (100) |
| SOUND-C1 | NCT01132313 | Treatment-naïve | 18/18 (100) | 18/18 (100) |  | 13/13 (100) | 13/13 (100) |
| SOUND-C2  SOUND-C3 | NCT01132313 | Treatment-naïve | 211/211 (100) | 211/211 (100) |  | 233/233 (100) | 233/233 (100) |
| HCVerso1  HCVerso2 | NCT01732796  NCT01728324 | Treatment-naïve | NA | NA |  | 950/954 (99.6) | 952/954 (99.7) |
|  |  | **TOTAL** | 229/229 (100) | 292/292 (100) |  | 1196/1200 (99.7) | 1264/1266 (99.8) |

^a^NS3 amino acids 1–631 + NS4A amino acids 1–54. ^b^NS5B amino acids 1–591. ^c^N = number of patients in the full analysis set. ^d^Resistance analyses for study 1241.2 have been previously described [2]. ^e^Resistance analyses for study 1241.7 have been previously described [7].

GT, genotype; HCV, hepatitis C virus; NA, not applicable for phase 1b studies of deleobuvir.

## Supplementary Table C. Numbers of non-SVR12 patients treated with faldaprevir, deleobuvir, and RBV who had baseline and post-baseline HCV sequences.

| **Trial** | **Trial identifier(s)** | **Patient**  **population** | **GT-1a** | |  | **GT-1b** | |
| --- | --- | --- | --- | --- | --- | --- | --- |
|  |  |  | **NS3/4A^a^ n/N^c^ (%)** | **NS5B^b^  n/N^c^ (%)** |  | **NS3/4A^a^ n/N^c^ (%)** | **NS5B^b^  n/N^c^ (%)** |
| SOUND-C2^d^  SOUND-C3 | NCT01132313 | Treatment-naïve | 113/211 (54) | 113/211 (54) |  | 41/233 (18) | 40/233 (18) |
| HCVerso1  HCVerso2 | NCT01732796  NCT01728324 | Treatment-naïve | NA | NA |  | 175/954 (18) | 178/954 (19) |
|  |  | **TOTAL** | 113/211 (54) | 113/211 (54) |  | 216/1187 (18)^e^ | 218/1187 (18)^e^ |

^a^NS3 amino acids 1–631 + NS4A amino acids 1–54. ^b^NS5B amino acids 1–591. ^c^N = number of patients in the full analysis set.

^d^Does not include patients in the RBV-free treatment group. ^e^220 patients had at least one sequence (NS3/4A or NS5B) available.

GT, genotype; HCV, hepatitis C virus; NA, not applicable for phase 3 studies of GT-1b patients; RBV, ribavirin; SVR12, sustained virologic response 12 weeks after the end of treatment.

## Supplementary Table D. Impact of baseline polymorphisms on SVR12 rates following treatment with faldaprevir, deleobuvir, and RBV among GT-1b patients in phase 2 studies.

| **Baseline variant** | **With variant^a^** | | **Without variant^b^** | |  |
| --- | --- | --- | --- | --- | --- |
|  | **N** | **SVR12,  n (%)** | **N** | **SVR12,  n (%)** | **Fisher's  *P* value** |
| GT-1b NS5B A421V | 12 | 11 (91.7) | 193 | 140 (72.5) | 0.1898 |
| GT-1b NS5B V499A | 32 | 25 (78.1) | 173 | 126 (72.8) | 0.6638 |
| GT-1b NS3 T344I | 29 | 22 (75.9) | 176 | 129 (73.3) | 1.0000 |

Phase 2 data from SOUND-C2 (RBV-containing arms only) and SOUND-C3.

^a^‘With variant’ includes only the single amino acid variant of interest and does not include wild-type, other variants, or mixtures of the variant of interest with wild-type or other amino acids.

^b^‘Without variant’ includes wild-type and all other amino acid variants or mixtures detected.

GT, genotype; RBV, ribavirin; SVR12, sustained virologic response 12 weeks after treatment.

## Supplementary Table E. Treatment-emergent variants at NS5B codons 389, 415, and 390 or GT-1b NS3 codon 344 in patients who did not achieve SVR12 with faldaprevir, deleobuvir, and RBV

| **GT-1a NS5B** | | **BT (N=73)** | **Other^a^ (N=25)** | **Relapse (N=15)** | **Total (N=113)** |
| --- | --- | --- | --- | --- | --- |
| **WT codon** | **AA variant** | **n (%)** | **n (%)** | **n (%)** | **n (%)** |
| T389 | I/T | 1 (1.4) | 0 (0) | 0 (0) | 1 (0.9) |
| T390 | None | 73 (100) | 25 (100) | 15 (100) | 113 (100) |
| F415 | F/Y | 1 (1.4) | 0 (0) | 0 (0) | 1 (0.9) |
|  |  |  |  |  |  |
| **GT-1b NS5B** | | **BT (N=92)** | **Other^a^ (N=73)** | **Relapse (N=53)** | **Total (N=218)** |
| **WT codon** | **AA variant** | **n (%)** | **n (%)** | **n (%)** | **n (%)** |
| T389 | None | 92 (100) | 73 (100) | 53 (100) | 218 (100) |
| T390 | I | 1 (1.1) | 0 (0) | 0 (0) | 1 (0.5) |
|  | I/T | 4 (4.3) | 0 (0) | 0 (0) | 4 (1.8) |
|  | V | 0 (0) | 0 (0) | 1 (1.9) | 1 (0.5) |
| Y415 | None | 92 (100) | 73 (100) | 53 (100) | 218 (100) |
|  |  |  |  |  |  |
| **GT-1b NS3** | | **BT (N=92)** | **Other^a^ (N=70)** | **Relapse (N=54)** | **Total (N=218)** |
| **WT codon** | **AA variant** | **n (%)** | **n (%)** | **n (%)** | **n (%)** |
| T344 | A | 1 (1.1) | 0 (0) | 1 (1.9) | 2 (0.9) |
|  | A/T | 0 (0) | 0 (0) | 1 (1.9) | 1 (0.5) |
|  | I | 0 (0) | 1 (1.4) | 0 (0) | 1 (0.5) |
|  | I/T | 0 (0) | 1 (1.4) | 0 (0) | 1 (0.5) |
|  | I/V | 0 (0) | 1 (1.4) | 0 (0) | 1 (0.5) |
|  | V | 0 (0) | 0 (0) | 2 (3.7) | 2 (0.9) |

Data pooled from SOUND-C2 (RBV-containing arms only), SOUND-C3, and HCVerso1 and 2.

Only patients with baseline and at least one post-baseline sequence are included in the table.

Table is based on a ‘post-baseline all’ analysis pooling emerging variants detected among all post-baseline visits per patient.

^a‘^Other’ category for phase 2 includes non-response at week 6, lack of end of treatment response, SVR12 missing, premature discontinuation. The ‘Other’ category for phase 3 includes premature discontinuation, lack of end of treatment response or lost to follow-up, and excludes discontinuation during placebo treatment.

AA, amino acid; BT, breakthrough; GT, genotype; RBV, ribavirin; SVR12, sustained virologic response 12 weeks after treatment; WT, wild-type.

## Supplementary Table F. Treatment-emergent RAVs detected in patients without SVR12 following RBV-free treatment with faldaprevir plus deleobuvir in study SOUND-C2.

| **GT-1a** | | **BT**  **(N=10)** | **Other^a^**  **(N=3)** | **Relapse**  **(N=1)** | **Total**  **(N=14)** |
| --- | --- | --- | --- | --- | --- |
| **NS3 RAV** | **NS5B RAV** | **n (%)** | **n (%)** | **n (%)** | **n (%)** |
| R155K, K/R | None | 0 (0) | 3 (100) | 1 (100) | 4 (28.6) |
| R155K | P495L, L/P | 6 (60) | 0 (0) | 0 (0) | 6 (42.9) |
| R155K | P495L/P/S | 2 (20) | 0 (0) | 0 (0) | 2 (14.3) |
| R155K/R | P495P/T | 1 (10) | 0 (0) | 0 (0) | 1 (7.1) |
| R155K/R+D168D/V | P495L | 1 (10) | 0 (0) | 0 (0) | 1 (7.1) |
|  |  |  |  |  |  |
| **GT-1b** | | **BT**  **(N=8)** | **Other**  **(N=2)** | **Relapse**  **(N=1)** | **Total**  **(N=11)** |
| **NS3 RAV** | **NS5B RAV** | **n (%)** | **n (%)** | **n (%)** | **n (%)** |
| None | None | 0 (0) | 2 (100) | 1 (100) | 3 (27.3) |
| D168V | P495L | 4 (50) | 0 (0) | 0 (0) | 4 (36.4) |
| D168V | P495L/Q | 1 (12.5) | 0 (0) | 0 (0) | 1 (9.1) |
| D168V | P495P/Q | 1 (12.5) | 0 (0) | 0 (0) | 1 (9.1) |
| R155K | P495L/P/S | 1 (12.5) | 0 (0) | 0 (0) | 1 (9.1) |
| D168V+S61L/S | P495L/Q | 1 (12.5) | 0 (0) | 0 (0) | 1 (9.1) |

^a^‘Other’ category for phase 2 includes non-response at week 6, lack of end of treatment response, SVR12 missing, premature discontinuation. The ‘Other’ category for phase 3 includes premature discontinuation, lack of end of treatment response or lost to follow−up, and excludes discontinuation during placebo.

BT, breakthrough; GT, genotype; RAV, resistance-associated variant; RBV, ribavirin; SVR12, sustained virologic response 12 weeks after treatment.

## Supplementary Table G. Treatment-emergent RAVs detected in samples from patients without SVR24 following treatment with faldaprevir, plus deleobuvir,^a^ and plus RBV followed by faldaprevir + PegIFN/RBV in study SOUND-C1.

| **Patient** | **HCV**  **GT** | **Deleobuvir treatment  group** | **SVR24 outcome** | **Emergent NS3 RAV^b^** | **Emergent NS5B RAV^c^** | **Baseline RAV** |
| --- | --- | --- | --- | --- | --- | --- |
| A | 1a | 400 mg TID | BT^c^ | R155K | P495L | NS5B A421A/V |
| B | 1a | 400 mg TID | Relapse | R155K | None | NS5B A421V |
| C | 1a | 400 mg TID | Relapse | R155K | None | None |
| D | 1a | 400 mg TID | Responder^e^ | R155K | n.a. | None |

**^a^**All these patients were in the TID deleobuvir treatment group.

**^b^**NS3 RAVs: variants at codons 155, 156, and/or 168.

**^c^**NS5B RAVs: variants at codons 421, 495, 496, and/or 499.

^d^BT during the 4-week IFN-free phase.

^e^Patient experienced a transient increase in HCV RNA of 0.7 log_10_ IU/mL during the 4-week IFN-free phase, but achieved SVR24 as previously described [12].

BT, breakthrough; GT, genotype; HCV, hepatitis C virus; IFN, interferon; n.a., not available (sequence could not be obtained); RAV, resistance-associated variant; RBV, ribavirin; SVR24, sustained virologic response 24 weeks after treatment; Peg, pegylated; TID, three-times daily.

## Supplementary Table H. Susceptibility of NS3 site-directed mutants to faldaprevir *in* vitro.

|  | **GT-1a NS3** | |  | **GT-1b NS3** | |
| --- | --- | --- | --- | --- | --- |
| **Site-directed mutant** | **%RC ± SD (n)** | **FDV EC_50_ FC ± SD (n)** |  | **%RC ± SD (n)** | **FDV EC_50_ FC ± SD (n)** |
| R155G | 32 ± 6 (10) | 150 ± 40 (7) |  | 34 ± 8 (11) | 190 ± 40 (8) |
| R155Q | n.a. | n.a. |  | 61 ± 23 (10) | 85 ± 37 (5) |
| R155K | **190 ± 80 (17)** | **190 ± 60 (14)** |  | 150 ± 50 (9) | 210 ± 100 (5) |
| R155S | 78 ± 16 (11) | 140 ± 40 (7) |  | n.a. | n.a. |
| R155T | 130 ± 50 (7) | 140 ± 30 (6) |  | n.a. | n.a. |
| R155W | n.a. | n.a. |  | 29 ± 9 (9) | 160 ± 20 (6) |
| A156S | n.a. | n.a. |  | 290 ± 250 (16) | 1.2 ± 0.2 (10) |
| A156T | n.a. | n.a. |  | 150 ± 90 (14) | 230 ± 80 (8) |
| A156V | n.a. | n.a. |  | 66 ± 38 (9) | 140 ± 40 (8) |
| D168A | 62 ± 19 (9) | 380 ± 60 (6) |  | 55 ± 22 (8) | 400 ± 220 (4) |
| D168E | n.a. | n.a. |  | 12 ± 4 (8) | 19 ± 3 (3) |
| D168F | n.a. | n.a. |  | 8.6 ± 3.2 (12) | 700 ± 160 (7) |
| D168G | n.a. | n.a. |  | 85 ± 15 (9) | 86 ± 12 (6) |
| D168H | n.a. | n.a. |  | 36 ± 10 (12) | 330 ± 70 (7) |
| D168I | n.a. | n.a. |  | 1.1 ± 0.4 (12) | 1200 ± 300 (6) |
| D168N | 93 ± 58 (7) | 5.7 ± 2.0 (6) |  | 83 ± 24 (10) | 5.8 ± 2.0 (6) |
| D168T | n.a. | n.a. |  | 160 ± 120 (12) | 580 ± 130 (7) |
| D168V | 2.8 ± 1.0 (10) | 810 ± 130 (4) |  | **11 ± 11 (15)** | **870 ± 230 (8)** |
| D168Y | n.a. | n.a. |  | 4.5 ± 1.4 (13) | 860 ± 230 (8) |
| R155K+D168E | 90 ± 19 (9) | 290 ± 70 (6) |  | n.a. | n.a. |
| R155G+D168N | n.a. | n.a. |  | 100 ± 20 (10) | 220 ± 50 (6) |
| R155Q+D168N | n.a. | n.a. |  | 59 ± 14 (10) | 120 ± 30 (7) |
| R155T+D168N | 88 ± 50 (9) | 220 ± 40 (6) |  | n.a. | n.a. |
| R155K+D168V | n.a. | n.a. |  | 26 ± 12 (9) | 190 ± 20 (4) |
| R155Q+D168V | n.a. | n.a. |  | 6.6 ± 3.7 (11) | 48 ± 14 (7) |

**Bold** text indicates variants that were detected in ≥10% of treated non-SVR12 patients.

Mean %RC and EC_50_ FC calculated from multiple (n) intra-experimental values relative to wild-type comparator.

EC_50_, 50% effective concentration; FC, fold change relative to wild-type; FDV, faldaprevir; GT, genotype; n.a., not assessed; RC, replicative capacity (% of wild-type); SD, standard deviation; SVR12, sustained virologic response 12 weeks after treatment.

## Supplementary Table I. Susceptibility of NS5B site-directed mutants to deleobuvir *in vitro*.

| **Site-directed**  **mutant** | **GT-1a NS5B** | |  | **GT-1b NS5B** | |
| --- | --- | --- | --- | --- | --- |
|  | **%RC  ± SD (n)** | **DBV EC_50_ FC ± SD (n)** |  | **%RC  ± SD (n)** | **DBV EC_50_ FC ± SD (n)** |
| T389S | 13 ± 2 (6) | 2.5 ± 0.1 (4) |  | n.a. | n.a. |
| A421T | 25 ± 2 (3) | 2.2 ± 0.5 (3) |  | n.a. | n.a. |
| A421V | **21 ± 4 (7)** | **3.2 ± 0.6 (4)** |  | 46 ± 14 (10) | 2.5 ± 0.6 (6) |
| A421V+P495L | **9.6 ± 1.6 (7)** | **150 ± 53 (4)** |  | 6.6 ± 2.2 (10) | 1300 ± 460 (3) |
| P495L | **33 ± 26 (13)** | **47 ± 15 (9)** |  | **12 ± 4 (5)** | **640 ± 140 (4)** |
| P495Q | 27 ± 19 (10) | 70 ± 15 (7) |  | 12 ± 6 (3) | 770 ± 400 (3) |
| P495S | 24 ± 13 (10) | 23 ± 3 (6) |  | 8.7 ± 1.2 (3) | 160 ± 79 (3) |
| P495T | 3.7 ± 1.4 (10) | 73 ± 35 (6) |  | 16 ± 5 (3) | 450 ± 240 (3) |
| P496A | 15 ± 3 (8) | 4.6 ± 0.7 (5) |  | 12 ± 2 (3) | 19 ± 2 (3) |
| P496S | 9.6 ± 3.3 (7) | 3.3 ± 0.9 (4) |  | 15 ± 1 (2) | 12 ± 4 (3) |
| P496L | n.a. | n.a. |  | 6 ± 1.6 (4) | 16 ± 1 (4) |
| P495S+P496S | n.a. | n.a. |  | 4.5 ± 0.7 (2) | 410 ± 140 (3) |
| V499A | n.a. | n.a. |  | 39 ± 13 (4) | 6.4 ± 0.6 (3) |

**Bold** text indicates variants that were detected in ≥10% of treated non-SVR12 patients.

Mean %RC and EC_50_ FC calculated from multiple (n) intra-experimental values relative to wild-type comparator.

DBV, deleobuvir; EC_50_, 50% effective concentration; FC, fold-change; GT, genotype; n.a., not assessed; RC, replicative capacity; SD, standard deviation; SVR12, sustained virologic response 12 weeks after treatment.

## Supplemental references

[1] Berger KL, Triki I, Cartier M, Marquis M, Massariol MJ, Bocher WO, et al. Baseline hepatitis C virus (HCV) NS3 polymorphisms and their impact on treatment response in clinical studies of the HCV NS3 protease inhibitor faldaprevir. Antimicrob Agents Chemother 2014;58:698-705.

[2] Larrey D, Lohse AW, Trepo C, Bronowicki JP, Arasteh K, Bourliere M, et al. Antiviral effect, safety, and pharmacokinetics of five-day oral administration of Deleobuvir (BI 207127), an investigational hepatitis C virus RNA polymerase inhibitor, in patients with chronic hepatitis C. Antimicrob Agents Chemother 2013;57:4727-4735.

[3] Delang L, Froeyen M, Herdewijn P, Neyts J. Identification of a novel resistance mutation for benzimidazole inhibitors of the HCV RNA-dependent RNA polymerase. Antiviral Res 2012;93:30-38.

[4] Young KC, Lindsay KL, Lee KJ, Liu WC, He JW, Milstein SL, et al. Identification of a ribavirin-resistant NS5B mutation of hepatitis C virus during ribavirin monotherapy. Hepatology 2003;38:869-878.

[5] Bartels DJ, Tigges AM, Sullivan JC, Henshaw J, Jiang M, Zhang EZ. Enrichment of the NS5B polymerase variant F415Y following failure of ribavirin containing regimens in patients with subtype 1a HCV. 6th International Workshop on Hepatitis C 2011;Boston, MA, USA. June 23-24:R12-1991.

[6] Ward CL, Dev A, Rigby S, Symonds WT, Patel K, Zekry A, et al. Interferon and ribavirin therapy does not select for resistance mutations in hepatitis C virus polymerase. J Viral Hepat 2008;15:571-577.

[7] Larrey D, Lohse AW, de Ledinghen V, Trepo C, Gerlach T, Zarski JP, et al. Rapid and strong antiviral activity of the non-nucleosidic NS5B polymerase inhibitor BI 207127 in combination with peginterferon alfa 2a and ribavirin. J Hepatol 2012;57:39-46.

[8] Zeuzem S, Soriano V, Asselah T, Bronowicki J-P, Lohse AW, Müllhaupt B, et al. Faldaprevir and Deleobuvir for HCV Genotype 1 Infection. N Engl J Med 2013;369:630-639.

[9] Zeuzem S, Dufour JF, Buti M, Soriano V, Buynak RJ, Mantry P, et al. Interferon-free treatment of chronic hepatitis C with faldaprevir, deleobuvir and ribavirin: SOUND-C3, a Phase 2b study. Liver Int 2015;35:417-421.

[10] Sarrazin C, Castelli F, Puoti M, Shiffman M, Protescu L, Forns X, et al. HCVerso1: A phase III study of faldaprevir (FDV) plus deleobuvir (DBV) and ribavirin (RBV) for chronic HCV genotype (GT)-1b infection in treatment-naïve patients. Hepatol Res 2014;60:1150A (Abstract 1959).

[11] Nelson D, Andreone P, Colombo M, Calinas F, Oliveira A, Delwaide J, et al. HCVerso2: A phase III study of faldaprevir (FDV) plus deleobuvir (DBV) and ribavirin (RBV) for chronic HCV genotype (GT)-1b infection in treatment-naïve patients including those ineligible for pegylated interferon (PegIFN). Hepatology 2014;60:1150A (Abstract 1966).

[12] Zeuzem S, Asselah T, Angus P, Zarski JP, Larrey D, Mullhaupt B, et al. Faldaprevir (BI 201335), deleobuvir (BI 207127) and ribavirin oral therapy for treatment-naive HCV genotype 1: SOUND-C1 final results. Antivir Ther 2013;18:1015-1019.
